# Sign Language Recognition Using Wearable Electronics: Implementing k-Nearest Neighbors with Dynamic Time Warping and Convolutional Neural Network Algorithms

**DOI:** 10.3390/s20143879

**Published:** 2020-07-11

**Authors:** Giovanni Saggio, Pietro Cavallo, Mariachiara Ricci, Vito Errico, Jonathan Zea, Marco E. Benalcázar

**Affiliations:** 1 Department of Electronic Engineering, University of Rome “Tor Vergata”, Via Politecnico 1, 00133 Rome, Italy; saggio@uniroma2.it (G.S.); maryclair_91@hotmail.it (M.R.); 2 Data Analysis Group, MathWorks, Matrix House, Cambridge Business Park, Cambridge CB4 0HH, UK; p.cavallo85@gmail.com; 3 Department of Informatics and Computer Science, Escuela Politécnica Nacional, Quito 170517, Ecuador; marco.benalcazar@epn.edu.ec (M.E.B.); z_tjalezea@yahoo.com (J.Z.)

**Keywords:** wearable electronics, gesture recognition, sign language, sensory glove, IMU, classifiers

## Abstract

We propose a sign language recognition system based on wearable electronics and two different classification algorithms. The wearable electronics were made of a sensory glove and inertial measurement units to gather fingers, wrist, and arm/forearm movements. The classifiers were k-Nearest Neighbors with Dynamic Time Warping (that is a non-parametric method) and Convolutional Neural Networks (that is a parametric method). Ten sign-words were considered from the Italian Sign Language: cose, grazie, maestra, together with words with international meaning such as google, internet, jogging, pizza, television, twitter, and ciao. The signs were repeated one-hundred times each by seven people, five male and two females, aged 29–54 y ± 10.34 (SD). The adopted classifiers performed with an accuracy of 96.6% ± 3.4 (SD) for the k-Nearest Neighbors plus the Dynamic Time Warping and of 98.0% ± 2.0 (SD) for the Convolutional Neural Networks. Our system was made of wearable electronics among the most complete ones, and the classifiers top performed in comparison with other relevant works reported in the literature.

## 1. Introduction

Generally speaking, standardized signs or gestures improve communication [1], as it occurs in army and aircraft security scenarios [2], or improve interaction, as it occurs in human–machine systems [3,4,5] and in tele-control [6], or improve efficiency, as it occurs in surgery [7], just to mention a few. Specifically, signs that are structured with syntax, semantics, grammar, pragmatics morphology, and phonology become “sign languages” [8], which allow us the expression of thoughts and feelings, in the same way as a “natural language” can do.

Sign languages spread worldwide. This occurs especially because of a large amount of hearing disabilities, suffered by more than 466 million people, as pointed out by the World Health Organization (WHO, 2018) [9]. Sign languages are used by muted, deaf, aurally challenged, and hear impaired people, along as with their relatives and educators. Unfortunately, most of the people are not used to, or do not know, sign languages so their communication with hearing and speaking impaired people is quite difficult. Fortunately, more and more technologies focus on solving this challenging issue, measuring signs and assigning to each gesture the appropriate meaning.

Signs consist of sequences of movements of the upper limbs [10]. Therefore, we have to focus on the measures of these movements, and rests, of each finger, of the wrist and of the arm and forearm too [11]. Such measures can be performed by means of two main approaches, namely image-based and sensor-based methods [8,12]. In particular, the image-based approach demands less-expensive technology, but appropriate light and unobstructed-view conditions, and usually demand of high computational resources. On the other hand, the sensor-based approach requires the signers to wear devices, which can be of some discomfort, but the user is not limited by the scene conditions, since there is no need to stay in front of a camera.

For this work, we decided the use of the sensor-based approach. In particular, we used wearable electronics composed of a homemade sensory glove, used for measuring the movements of the fingers, and inertial measurement units (IMUs), used for measuring the movements of the wrist, forearm, and arm. The potential discomfort of the signers wearing these sensors is minimized, because we built the sensory glove according to the needs and requests of the users, and applied the IMUs with simple Velcro self-gripping straps.

Our research focused on the Italian sign language (Lingua Italiana dei Segni, LIS), that is the Italian one, but our methodology applies to whatever sign language. Our dataset consisted of seven thousand instances of ten gestures measured on seven signers, who performed one hundred instances of each gesture.

For the sign recognition, we considered two classifiers: one based on the k-Nearest Neighbors (k-NN) plus the Dynamic Time Warping (DTW) algorithms (i.e., a non-parametric method), and the other based on Convolutional Neural Networks (CNNs) (i.e., a parametric method). In particular, the k-NN method is applied for the recognition of gestures that may vary in speed, but having similar patterns in the shape of the temporal sequences, whereas the CNN has been more and more proving state of the art accuracies for different kind of signals (in particular for 1D, images, and video).

From the side of measurements, our efforts were particularly devoted to implement a full set of wearable devices, measuring both hand/forearms/arms and fingers of the dominant hand, to realize a dataset with an important number of gesture repetitions (100), and to enroll non-native gesture-speakers, so as to rely on low-repeatability gestures in order to stress the classification algorithms. From the side of classification, we adopted two different approaches, competing or even better performing related works on sign language recognition.

## 2. Related Works

In the scientific literature, the works that adopt a sensor-based approach use a sensory glove with embedded flex sensors, or inertial measurement units (IMUs), or both [13]. Flex sensors are used for measuring flexion and extension of the fingers’ joints [14,15]; IMUs are used for measuring the accelerations and rotations of the palm/wrist and/or the arm/forearm. The number of involved signers, the number of measured signs, and the number of sign repetitions can vary from work to work. Moreover, a number of different classification algorithms have been reported in the literature, underlying the challenging issues to be solved towards the optimal solution for sign language recognition. Within this frame, in the remainder of this section, we present some relevant works for comparison purposes.

Mohandes et al. [16] used the PowerGlove, which provides hand’s space position, wrist roll, and four bending finger movements [17]. The glove was used to repeat 10 signs for 20 times. The adopted support vector machine (SVM) algorithm reached an accuracy greater than 90% ± 10(SD).

Mohandes and Deriche [18] measured finger movements using two CyberGloves, with 22 embedded sensors each, and hand movements using the so-called 6-Degrees of Freedom (DOF) “Flock of Birds” device. One signer performed 20 repetitions of 100 two-handed signs. The data dimensionality was reduced by means of Linear Discriminant Analysis (LDA), and the classification was based on the minimum distance (MD) algorithm, reaching an accuracy of 96.2% ± 0.78 (SD).

Two DG5-VHand data gloves, both equipped with five flex sensors and a three-axes accelerometer, were adopted by Tubaiz et al. [19]. Those two gloves measured gestures performed by one signer, who repeated ten times 40 sentences, later divided into 80 words by means of manual labeling separation. With the use of a Modified k-Nearest Neighbors classifier (Mk-NN), researchers obtained the best result as 82% ± 4.88 (SD).

Abualola et al. [20] used a glove equipped with six IMUs to measure fingers and palm movements of nine participants, while signing 24 static letters, one sample per letter per user. Linear Discriminant Analysis (LDA) was applied to reduce the data complexity later analyzed by the Euclidean distance-based classification algorithm, resulting with an accuracy of 85%.

Hernandez et al. [21] adopted a technology based on the “AcceleGlove” and a two-link arm skeleton. Their database consisted of data coming from 17 signers who repeated 30 times one-hand gestures. The classification was based on Conditional Template Matching (CTM) and the resulting recognition rate was equal to 98%.

Lu et al. [22] used the “YoBu” sensory glove, made of eighteen low-cost inertial and magnetic measurement units. This sensor was used to acquire both arm and hand motions, simultaneously. The glove acquired data of 10 types of static gestures. The classification was based on Extreme Learning Machine kernel (ELM-kernel) and on Support Vector Machine (SVM) algorithms, with classification accuracies of 89.59% and 83.65%, respectively.

A work done by Saengsri et al. [23] measured signs by means of a sensory glove and a motion tracker. The first was the “5DT Data Glove 14 Ultra”, with 14 sensors for measuring both flexures and abductions of the fingers. The latter provided 3D spatial coordinates of the hand. The dataset consisted of 16 signs, 4 samples for each sign made by a professional signer. The adopted Elman Back Propagation Neural Network (ENN) algorithm resulted with an accuracy of 94.44%.

Silva et al. [24] used a sensory glove, which was equipped with a flex sensor, a gyroscope and an accelerometer attached on the distal phalanx of each finger. The signs were alphabet characters, measured 100 times each. An Artificial Neural Network (ANN) reached 95.8% of recognition accuracy.

Although the aforementioned papers demonstrate important efforts to solve the problem of sign language recognition, the optimal solution remains a challenging problem. This is because there is a lack in defining the best measurement technology and the best classifier, all having advantages and drawbacks too.

We present two different classification algorithms, both competing or even better performing similar literature works. The algorithm based on convolutional neural networks is well suited for training sets from few to thousands, while the algorithm based on the k-nearest neighbors, combined with dynamic time warping, is a good option for scenarios with training sets made of examples in the order of dozens or hundreds.

This work explores possibilities gathered from a double technology measurement approach (a sensory glove plus IMUs), from a huge number of repetitions (100) of each gesture, and from a two-model classification approach (k-NN with DTW and CNN algorithms). In addition, given that native signers sign in highly repeatable manner, we adopted language signs performed by trainees (with lower repetition ability) in order to evidence the robustness of the system, if any.

## 3. Materials and Methods

**Figure 1:**
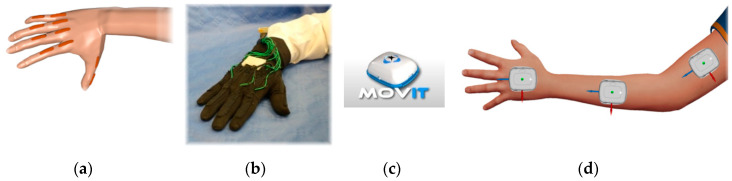
(**a**) Arrangement of the ten flex sensors on top of the carpal–metacarpal and metacarpal–phalangeal joints of the fingers. (**b**) The sensory glove equipped with the ten flex sensors, singularly hosted in one pocket each. (**c**) The inertial measurement units termed Movit. (**d**) Arrangement of the inertial measurement units (IMUs) on the dorsal aspect of the hand, on the forearm and arm, for both of the upper limbs.

Our wearable electronics (simply called wearables hereafter) is composed of a sensory glove and IMUs. In particular, the sensory glove allows measuring the movements of the fingers’ joints of the dominant hand (specifically the right one) for every signer (Figure 1a,b), and the IMUs allow measuring the spatial arrangements of the upper limbs (Figure 1c,d).

**Figure 2:**
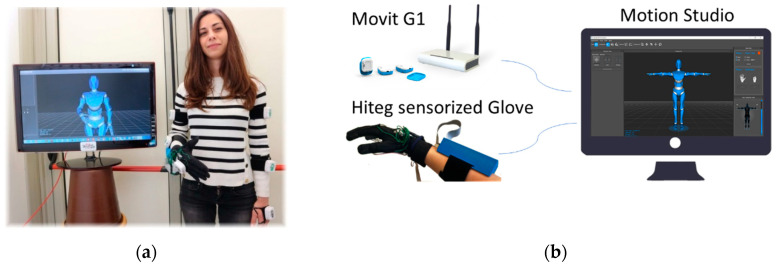
(**a**) A signer with the sensory glove and the six IMUs on the hand/forearm/arm. (**b**) Block diagram of the system: an avatar reproduces gestures on a computer screen, to visually control that the correct data flow from the sensors; the software manages the data stream and the synchronization of the two system.

In general, for the most part of sign languages (and in particular for the here adopted Italian one), the meaning concerns the movements of the fingers of dominant hand, the non-dominant one acting symmetrically or to intensify the meaning only. Therefore, here we adopted a single glove for the dominant hand. Figure 2 shows the overall system.

Data, wireless send via Zigbee protocol to a receiving unit, are stored on a personal computer (Intel i5, 8GB RAM) and reproduced on a screen via avatar (Figure 2).

### 3.1. Sensory Glove

We adopted an indigenously developed sensory glove, termed Hiteg Glove, in order to take into account the requests of comfort and usability evidenced by the signers. Our sensory glove consists of a 70% Lycra and 30% cotton fabric with 10 slim pockets, each hosting one flex sensor [25,26]. An on-purpose hardware translates the analog data from the flex sensors into 12-bit digital values at a 40 Hz sampling frequency. The pockets were sewn onto the regions of the carpal–metacarpal and the metacarpal–phalangeal joints to measure the flexion/extension movements of all fingers.

### 3.2. IMUs

We used six inertial measurement units (IMUs), three for the left and three for the right upper limb, so as to measure the space arrangement and the movements of the wrists, arms, and forearms. The IMU, previously validated [27,28,29], is termed “Movit G1” (by Captiks Srl Rome, Italy) (Figure 1c), and hosts three-axial accelerometer, gyroscope and a compass (plus a barometer and a thermometer too, but not used for our purposes). The sampling rate for measurement can be set within 4–200 Hz, the acceleration within ±2 g to ±16 g (gravities), the angular velocity within ±250 dps to ±2000 dps (degree per seconds). Each IMU can store the measured data and/or provide the data to a unique receiver via a proprietary wireless protocol, or through a USB cable.

### 3.3. Calibration and Data Acquisition

The “Captiks Motion Studio” software store data and reproduce gestures by means of an avatar on a computer screen. The starting procedure consists of a calibration step both for the sensory glove and for the IMUs. In particular for the glove, on the hypothesis of a linear response of the bend sensors, it is requested to the wearer to place the hand completely open (flat) and completely closed (wrist), so that the software will interpolate all the intermediate angles of the joints of the fingers.

For the IMUs, a patented calibration procedure consists of placing the IMUs on a support turned on three orthogonal planes in sequence, and then wearer by the subject posed in a “T” condition (the arms parallel to the floor) to assign a starting plane to each sensor.

The software synchronizes all acquired data by adding timestamps. Thus, the LIS recognition can benefit from simultaneous, multiple sensors, multiple technologies, synchronized data collection, and real-time kinematic reconstruction. The open-source universal messaging ZeroMQ socket (http://zeromq.org/) is configured for transferring the streaming of the acquired data from Motion Studio to MATLAB^®^ and here saved in MATLAB tables. The proposed off-line study permits to identify the most appropriate method for real-time LIS word recognition, further improvable for the LIS consecutive sentence recognition. For this work, we selected 40 Hz sampling rate from the glove’s flex sensors and 200 Hz sampling rate for the IMUs. Delays in the signal chain resulted in an effective average of 37.5 Hz data rate from IMUs. Moreover, we selected ±2 g and ±2000 dps for the acceleration and angular degree scales, respectively.

### 3.4. Signers and Signs

Seven signers were recruited for the experiments, five males and two females aged 29–54 y ± 10.34 (SD). All signers were right-handed and with similar hand dimensions, so that a unique-size right-hand sensory glove was used.

The signers were asked to replicate, as accurately as possible, 10 common sign gestures, as showed by a professional language signer. The 10 signs were selected among the most used in LIS (the Italian Sign Language), such as ciao, cose (things), grazie (thanks), maestra (teacher), google, internet, jogging, pizza, TV, and twitter. Most of these 10 signs refers to words internationally known.

Each signer, in turn, wore the sensory glove and the six IMUs by means of Velcro straps, and performed signs when sitting on a chair in front of a table, with his/her right hand resting on the table. The time duration of each sign depended on the natural performance of the user, without any constraint. For each user and each sign, a continuous stream of data, generated by the electronic wearables, was stored on a personal computer. For a mere qualitative correct data flow control, an avatar replicated the signer’s movements (Figure 2).

## 4. Classifiers

We applied two different classification models on the same dataset: the first is a non-parametric method based on the k-Nearest Neighbors (k-NN) classifier and the Dynamic Time Warping (DTW) algorithm, and the second is based on Convolutional Neural Networks (CNNs) [30].

### 4.1. k-NN with DTW

*Data representation*: The data acquired when a user performs a gesture, or class, is represented as a tuple H = ($\underline{F},\bar{\underline{A}},\bar{\underline{W}},L$). The matrix $\underline{F}$ contains the data acquired by the flex sensors from the sensory glove, the hyper-matrices $\bar{\underline{A}}$ and $\bar{\underline{W}}$ contain the data from the accelerometers and the gyroscopes, respectively. The categorical variable *L* = 1, 2, …, *c*, with *c*∈${\mathbb{Z}}^{+}$, denotes the corresponding label of the signals $\underline{F},\;\bar{\underline{A}},\;\bar{\underline{W}}$.

The matrix $\underline{F}=(F^{\left(1\right)},\ldots ,F^{\left(i\right)},\ldots ,F^{\left(10\right)})$ is formed by 10 column vectors of the form $F^{\left(i\right)}={\left(f_{1}^{\left(i\right)},\ldots ,f_{n}^{\left(i\right)},\ldots ,f_{N}^{\left(i\right)}\right)}^{T}$∈$R^{N\times 1}$, with *i* = 1, 2, …, 10 and *N*∈${\mathbb{Z}}^{+}$. The value of $f_{n}^{\left(i\right)}$ represents the electric resistance, in ohms, measured at the *n*th instant in the *i*th flex sensor of the Hiteg Glove, at a sampling frequency of 40 Hz. The hypermatrix $\bar{\underline{A}}=({\underline{\bar{A}}}^{\left(1\right)},\ldots ,{\underline{\bar{A}}}^{\left(j\right)},\ldots ,{\bar{\underline{A}}}^{\left(6\right)})$∈$R^{M\times 6\times 3}$ is formed by the hypermatrices A¯_(j)=(Ax(j),Ay(j),Az(j))∈$R^{M\times 1\times 3}$, with *j* = 1, 2, …, 6 and *M*∈${\mathbb{Z}}^{+}$. The vector As(j)=(as1(j),…,asm(j),…,asM(j))T∈$R^{M\times 1}$ is the measurement returned by the *j*th inertial sensor of the Movit G1 in the *s*th coordinate axis, with *s*∈{*x*,*y*,*z*}. The value of asm(j) represents the acceleration, in fractions of gravity, measured at a sampling frequency of 37.5 Hz at the *m*th instant of time.

Similarly, the hypermatrix $\bar{\underline{W}}=({\underline{\bar{W}}}^{\left(1\right)},\ldots ,{\underline{\bar{W}}}^{\left(j\right)},\ldots ,{\bar{\underline{W}}}^{\left(6\right)})$∈$R^{M\times 6\times 3}$ corresponds to the angular velocity, in degrees per second, measured at a sampling frequency of 37.5 Hz, and it is formed by the hypermatrices W¯_(j)=(Wx(j),Wy(j),Wz(j))∈$R^{M\times 1\times 3}$. The column vector Ws(j)=(ws1(j),…,wsm(j),…,wsM(j))T∈$R^{M\times 1}$ contains the data of the *j*th inertial sensor in the *s*th coordinate axis, with *s*∈{x,y,z}.

The length *N* of the column vectors $F^{\left(i\right)}$ of the flex sensors and the length *M* of the vectors As(j) of the accelerometer and the vectors Ws(j) of the angular velocity are not equal. This is because of the difference in the sampling rate of each sensor: 40 Hz for the flex sensors and 37.5 Hz for the IMU sensors.

Datasets: The signs are grouped to form a dataset $ℋ=\left\{{ℋ}_{1},\ldots ,{ℋ}_{1},\ldots ,{ℋ}_{U}\right\}$, with *U*∈${\mathbb{Z}}^{+}$, where the example ℋ*_u_* = (${\underline{F}}_{u},{\bar{\underline{A}}}_{u},{\bar{\underline{W}}}_{u},L_{u}$) is the *u*th instance of a gesture labeled with *L_u_*, where *u* = 1, 2, …, *U*. Data were randomly split into a test set (with 20% of the original dataset) and a training set (with the remaining 80% of the original dataset).

Training and testing sets: A subset ${ℋ}_{1}^{v}$ from the set **ℋ** was used for training and the remaining subset $ℋ-{ℋ}_{1}^{v}$ was used for testing, where 0 < *v* < *U*, with *v*∈${\mathbb{Z}}^{+}$. Since the k-NN with DTW algorithm does not need any training (but only the adjustment of the number of neighbors used for the classification), here the term training refers to differentiate data used to find the *k*-nearest neighbors to the signal being classified, which comes from the testing set used to estimate the accuracy of the classification model. Therefore, we should be clear that.

Pre-processing: In this stage, we first normalized the amplitude and then filtered the noise of the time series contained in the column vectors of the examples ($\underline{F},\bar{\underline{A}},\bar{\underline{W}}$) from the set ${ℋ}_{1}^{v}$.

For the normalization, we used the function $ℒ:R^{N\times 1}\to R^{N\times 1}$ defined through the following equation:

(1) $$ℒ\left(V\right)=\frac{V-{\bar{\underline{V}}}_{\min}}{{\bar{\underline{V}}}_{\max}-{\bar{\underline{V}}}_{\min}},$$

In this equation, **V**∈{$\underline{F},\bar{\underline{A}},\bar{\underline{W}}$} denotes the vector to normalize and ℒ(V) denotes the normalized vector. The values of ${\bar{\underline{V}}}_{\max}$ and ${\bar{\underline{V}}}_{\min}$ are computed among the values of the element from the set {$\underline{F},\bar{\underline{A}},\bar{\underline{W}}$} that **V** takes. For example, if we want to normalize the values of the vector **F**^(*i*)^∈$\underline{F}$, being **V** = **F**^(*i*)^, the ${\bar{\underline{V}}}_{\max}$ and ${\bar{\underline{V}}}_{\min}$ are the maximum and minimum values, respectively, computed among all the values of the column vectors that form the set {${\underline{F}}_{1},\ldots ,{\underline{F}}_{v}$}. A similar process is applied for the case where **V** = As(j) and **V** = Ws(j). This normalization function ℒ is applied over all the time series of each example from the training set ${ℋ}_{1}^{v}$, so that each component of the new vectors is in the range [0,1]. Applying this normalization over all the instances (i.e., tuples) of the training set, we obtain the new set $ℒ\left({ℋ}_{1}^{v}\right)=\left\{{\left(ℒ\left({\underline{F}}_{u}\right),ℒ\left({\bar{\underline{A}}}_{u}\right),ℒ\left({\bar{\underline{W}}}_{u}\right),L_{u}\right)}_{u=1}^{v}\right\}$.

For filtering, we apply a low pass filter ψ to the normalized time series $ℒ\left({\bar{\underline{A}}}_{u}\right)$ and $ℒ\left({\bar{\underline{W}}}_{u}\right)$ of the inertial sensors only. The function ψ is a digital Butterworth filter [31] of 4th order, where the cutoff frequency is *f_c_* = 0.05 π*f*_s_, where *f*_s_ is the value of the sampling frequency in Hz. From this step, we obtain the set:

(2) $$\psi \left(ℒ\left({ℋ}_{1}^{v}\right)\right)=\left\{{\left(ℒ\left({\underline{F}}_{u}\right),\psi \left(ℒ\left({\bar{\underline{A}}}_{u}\right)\right),\psi \left(ℒ\left({\bar{\underline{W}}}_{u}\right)\right),L_{u}\right)}_{u=1}^{v}\right\}$$

Classification: The classification assigns a label to a gesture **X**∈${ℋ}_{v+1}^{U}$ of the testing set based on the *k*-Nearest Neighbors (k-NN) classifier and the Dynamic Time Warping (DTW) algorithm [32].

We computed a distance value *d*∈$R^{+}$ that represents the similarity between the unlabeled preprocessed gesture ψ(ℒ(X)), with X = ($\underline{F},\bar{\underline{A}},\bar{\underline{W}}$), and the 3 first elements of each tuple from the preprocessed training set $\psi \left(ℒ\left({ℋ}_{1}^{v}\right)\right)$. We used the DTW algorithm [33] to optimally align the corresponding channels of ψ(ℒ(X)) and each tuple from the set $\psi \left(ℒ\left({ℋ}_{1}^{v}\right)\right)$. For computing the distance *d* between ψ(ℒ(X)) and the three first elements of each tuple from the set $\psi \left(ℒ\left({ℋ}_{1}^{v}\right)\right)$, we first computed the DTW distance $d(F^{\left(i\right)})$ between the corresponding channels of the flex sensors (Equation (3)), the distance d(A(s)(j)) between the corresponding channels of the accelerometers (Equation (4)), and the distance d(W(s)(j)) between the corresponding channels of the angular velocity (Equation (5)).

(3) $$d\left(F^{\left(i\right)}\right)=DTW\left(ℒ\left(F^{\left(i\right)}\right),ℒ\left(F_{u}^{\left(i\right)}\right)\right),\;F^{\left(i\right)}\in X,$$

(4) d((s)A(j))=DTW(ψ(ℒ((s)A(j))),ψ(ℒ((s)Au(j)))), A(s)(j)∈X,

(5)  d((s)W(j))=DTW(ψ(ℒ((s)W(j))),ψ(ℒ((s)Wu(j)))), W(s)(j)∈X,

with *u* = 1, 2, …, *U*, where *U*∈${\mathbb{Z}}^{+}$ is the size of the training set.

For computing the total distance *d_u_* between ψ(ℒ(X)) and the *u*th tuple from the preprocessed training set $\psi \left(ℒ\left({ℋ}_{u=1}^{v}\right)\right)$, we summed all the DTW distances obtained using the Equations (3)–(5):

(6) du=∑i=110d(F(i)) +∑j=16(∑s∈{x,y,z}d(A(s)(j))+d(W(s)(j))).

The label *Y*, with *Y*∈{1, 2, …, *c*}, that the classifier returns for the unknown gesture X corresponds to the most voted label among the *k*-nearest neighbors in terms of the DTW distance, [34,35] found in the preprocessed training set $\psi \left(ℒ\left({ℋ}_{1}^{v}\right)\right)$.

For the kNN and DTW classification algorithm, we evaluated several implementations of DTW. The options that we tested range from evaluating the whole matrix of distances between the two signals to find the best path of alignment and more efficient implementations based on dynamic programming and window constraints of the space of search for the best path of alignment of the signals. The criteria that we used for evaluating different implementations of DTW was the gain in the classification accuracy versus the increase in the time of processing of the classification algorithm. For all the implementations that we tested, there was not a significant difference in terms of the classification accuracy, but there was an important difference in the time of processing. Based on this analysis, we chose the option based on dynamic programming with a window constraint in the space of search space of length 50 [36].

### 4.2. CNN

Datasets: In the same way as for the *k*-NN with the DTW algorithm, for the CNN classifier data were randomly split into a test set (20% of the original dataset) and a training set (the remaining 80%). The training set was further split into two subsets to create a validation set. As common practice in literature, we used a holdout validation scheme considering 20% of the training samples for model selection and hyper-parameters tuning and the remaining 80% for training the model.

Pre-processing: All signals were re-sampled to a fix number of 120 samples by means of a cubic spline interpolation using not-a-knot end conditions. Signals were further normalized, dividing each dimension by its maximum across the training set, using the normalization function $\;ℒ:R^{N\times 1}\to R^{N\times 1}$:

(7) $$ℒ\left(V\right)=\frac{V}{{\bar{\underline{V}}}_{\max}}.$$

**Figure 3:**
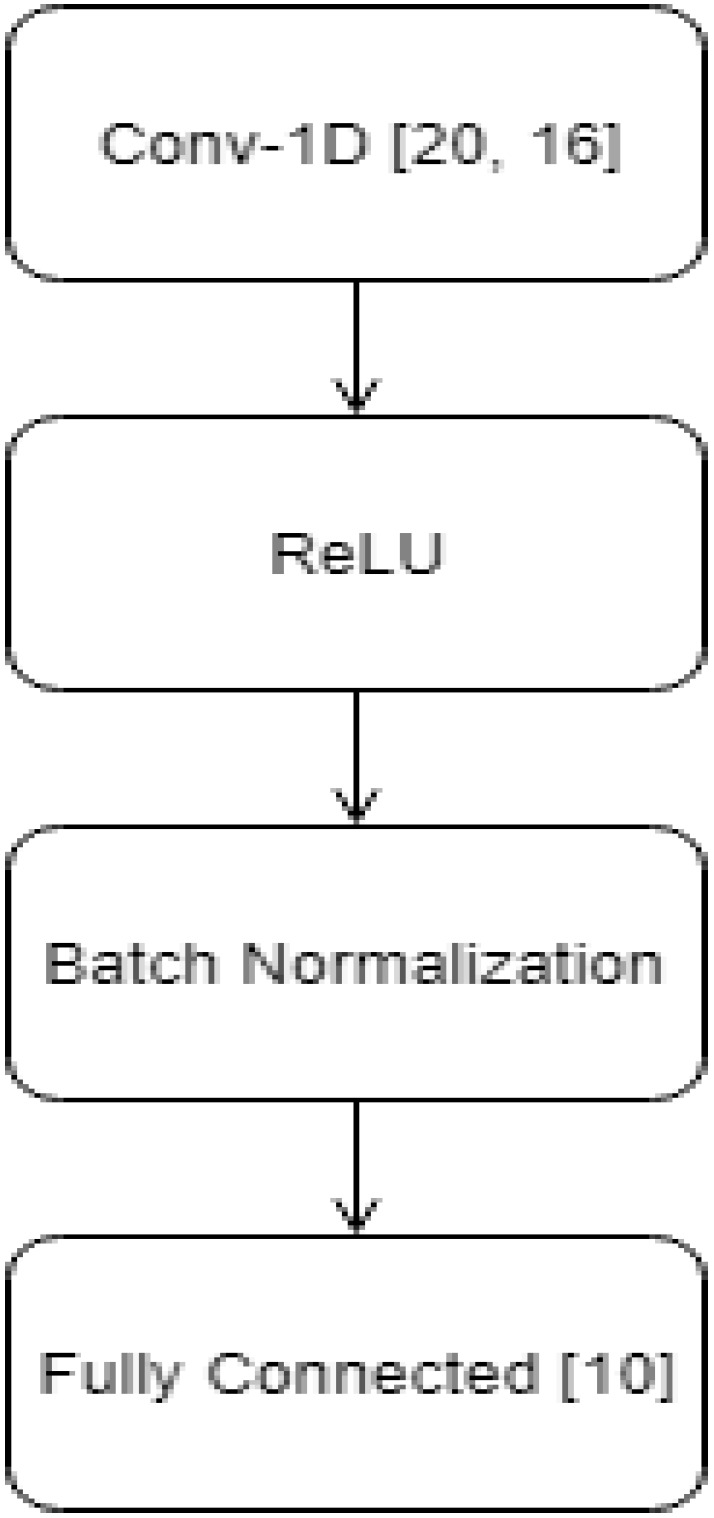
Convolutional Neural Network (CNN) architecture used in this work.

Network architecture: We tried several network architectures and hyper-parameter values for Convolutional Neural Networks (CNN) [30] including different numbers and complexity of the convolutional layers, various learning rates and mini-batch sizes. We also tried Long Short Term Memory (LSTM) networks [37]. Among all the architectures and the hyper-parameters values that we tried, the best validation accuracy was achieved by a CNN network with only one convolutional layer with 20 filters of size 16, ReLU activation and batch normalization [38], no pooling layers, and 16 × 16 sized kernel. A fully connected layer was then used to obtain the vector $Z=(Z_{1},\ldots ,Z_{c})\in R^{c\times 1}$, where *c* is the number of classes, being *c* = 10 in our case (Figure 3).

Training phase: We chose to optimize the cross-entropy loss function of the Matlab softmax function applied to the resulting vector Z together with L2 weights regularizes, where the *j*th component of the softmax is defined as follows:

(8) $$p_{j}=\frac{e^{Z_{j}}}{\sum_{k=1}^{c}e^{Z_{k}}},$$

with *j* = 1,…, *c*. The cross-entropy between the resulting vector of probabilities **p** = (*p*_1_, …, *p_c_*) predicted by the network and the expected one-hot encoding **Y** = (*Y*_1_, …, *Y_c_*) of the actual label *Y* of the gesture **X** is defined as follows:

(9) $$H(Y,p)=-\sum_{i=1}^{c}Y_{i}\times \log(p_{i}).$$

The CNN was trained using stochastic gradient descent with momentum, with a learning rate of 10^−4^ and a mini-batch size of 200. The training went on for 9600 iterations and was stopped when the validation loss did not increase for five consecutive times. This took a total time of 244 s using a Tesla K40 GPU and MATLAB^®^ with its Neural Network Toolbox.

## 5. Results and Discussion

For this work, we used a dataset composed of 10 different gestures, repeated 100 times each by 7 users, obtaining a dataset of *U* = 7000 tuples. This dataset ${ℋ}_{1}^{U}$ was divided into two subsets: one subset ${ℋ}_{1}^{5600}$ contains 80% of the examples for designing and training the classification models, and the other subset ${ℋ}_{5601}^{7000}$, 20% of ${ℋ}_{1}^{U}$, was used for testing the models. Before performing this partition, the elements of the original dataset were randomly shuffled.

### 5.1. Results with the k-NN and DTW Algorithm

**Figure 4:**
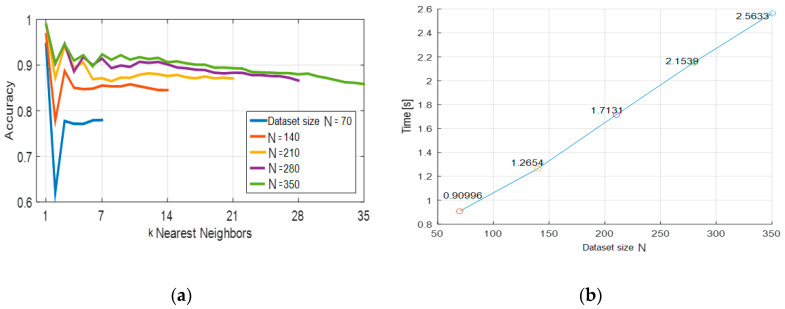
Related to the *k*-Nearest Neighbors (*k*-NN) and Dynamic Time Warping (DTW) classification algorithm: (**a**) Accuracy for different dataset sizes *N* and number of neighbors *k*; (**b**) Average time of classification versus the size *N* of the training set.

For defining the model composed of the *k*-NN classifier with DTW algorithm, we tested this model varying two parameters: the size *N* of the training set and the number *k* of neighbors. Each training set ${ℋ}_{1}^{N}$ was formed by selecting the first *N* elements from ${ℋ}_{1}^{5600}$, where *N* = 70, 140, 210, 280, 350, i.e., with an incremental step of 70 tuples, so that to balance the tuples added from each user. Moreover, the training set had the same number of tuples for each sign to recognize in order to be balanced. The number *k* of neighbors varies from 1 to *N*/7, where (*N*/7) ∈${\mathbb{Z}}^{+}$ is the number of tuples per gesture in the training set ${ℋ}_{1}^{N}$. It is worth noting that, for the classification using the k-NN with the DTW algorithm, we only used a small fraction of the training set in order to reduce the time of classification without reducing significantly the accuracy of the model. The accuracies reported in Figure 4a were computed by applying each model tested to all the tuples in the test set ${ℋ}_{5601}^{7000}$. Figure 4b shows the variation of the time of classification versus the size *N* of the training set. Each point of Figure 4b was obtained as the average of the times of classifying each tuple from the test set.

As Figure 4 shows, the accuracy of the classification model improves with the increase of the size *N* of the training set; however, the time required for training a model with a larger number of examples increases at a growth rate of approximately *O(N)*. Table 1 shows the confusion matrix related to the results obtained when the classification model was trained using *N* = 140 examples (2 repetitions per user for each gesture) and just one neighbor, *k* = 1, for the *k*-NN algorithm. It is worth mentioning that these settings correspond to a balance between a good accuracy and a low processing time. In the confusion matrix, the predicted gestures are in the rows, whereas the actual gestures are in the columns. The percentage shown in each cell, except for the cells of the last column and the last row, is obtained by diving the value that accompanies the percentage by the sum of the values of all the cells of the matrix, except for the last row and the last column. Therefore, the accuracy of each method (the last value of the main diagonal) is obtained by adding up the percentages along the main diagonal, except for the last value. The precisions (percentages are shown in the last column) are computed by dividing the value of the main diagonal, in each row, by the sum of the values of that row. The sensitivities (percentages shown in the last row) are computed by dividing the value of the main diagonal, in each column, by the sum of the values of that column.

Table 1 shows two main results related to the confusion: on the lower row the sensitivity rates (i.e., percentage of gestures correctly classified over the total of gestures performed for a given class), and on the right column the precision rates (i.e., percentage of gestures correctly classified over the total of gestures predicted for a given class). Three gestures reach 100% of sensitivity rate: jogging, pizza, and twitter. On the other hand, the worst sensitivity is for the gesture cose with 87.5%, while its precision is 100%. This means that every time that cose was predicted, it was correct, but many cose signs were not identified as such. Interestingly, the sign google has an opposite behavior, because its precision is just 89.4% (the worst among all precisions), but its sensitivity is 96.4%. Finally, the overall recognition accuracy of the k-NN with DTW model is 96.6%.

### 5.2. Results with the CNN Algorithm

Table 2 shows the classification accuracy obtained by the CNN classifier. The experiments were repeated 10 times using different random seeds for hyperparameters initialization and the mean accuracy is reported with results equal to 98% on the test set, 98.04% on the validation set, and 99.8% on the training set. Deeper architectures with more than one convolutional layer and pooling layers led to comparable if not worse results. This was somehow expected, since the small number of classes. Among all the architectures that gave us comparable results, we picked the smallest since we envision this application to run in real time and a smaller network would lead to faster performances. It is worth mentioning that the time performance of the training phase on GPU is also very fast.

### 5.3. Comparison of Results with Related Works

The relevant works about sign language recognition were already discussed in Section 2. Now, after reporting our results, we present in Table 3 a comparison of our work with the technologies and the classification accuracies of the works reviewed in this paper. According to this comparison, our method performed better than the other methods. As far as we know, we consider this as a very promising result.

## 6. Conclusions

The non-parametric model that combines the *k*-NN classifier with the DTW algorithm has good classification accuracy (96.6%) and its time complexity has a linear growth with the number of examples used to find the *k*-nearest neighbors for classification. The classification accuracy of this model improves with the increase of the number of training examples, but at the cost of increasing also the time of classification. Therefore, practical applications of sign language classification, especially in real time, using the *k*-NN and DTW model demand a tradeoff between accuracy and time of processing. Even though the time of classification is a big drawback for this model when we have a large dataset, its high accuracy has to be considered, especially for scenarios where the number of examples for the classification is limited. As it is usual, the computing time of this and other models depends highly on the hardware used to run the model. The *k*-NN and DTW model was tested on a desktop computer with an Intel^®^ Core™ i7-3770S processor (Intel Corporation, Santa Clara, CA, USA) and 4 GB of RAM. The relatively high classification accuracy and linear time complexity of the *k*-NN and DTW model suggest that it can be a good option for applications where the processing and storage capabilities are limited and the number of available data is in the order of the dozens.

The CNN parametric classifier performed with a very high accuracy value, quite close to 100%. We also tried to run inference of this model using a CPU, and we got up to 500 gestures per second on computer with an Intel^®^ Core™ i7-7700 processor ((Intel Corporation, Santa Clara, CA, USA) and 16 GB of RAM. This speed of processing means that inference in real time is possible using the CNN classifier.

The difference between the accuracies of both models tested in this work might occur because the CNN model captures better the distribution underlying the data of the ten gestures that we used here. However, with the increase of the number of training examples, the *k*-NN and DTW model might lead to similar or even better results than the CNN model at the cost of a higher time of processing. However, increasing the number of training examples will increase the time of training of the CNN, but its time of prediction will be fixed since the complexity of the CNN does not change with the increase of the size of the training set. This last feature of the CNN and the results obtained in this work evidence that this model is an interesting option for sign language classification in scenarios with many training examples.

We reported comparisons of our results with those from other relevant works published in the scientific literature. Although a perfect fit among all the works is almost impossible, since there are differences in terms of the types of wearables and signs, and number of signers and signs, the higher accuracies obtained in this work suggest how our approach can be valuable for sign recognition purposes. There are some major differences with respect to other studies. In particular, the number of used sensors, that constitute a complete set for full fingers, hands/arms/forearms joints analysis; the 100 gesture repetitions for each LIS sign from each signer, that constitute a comprehensive set of valuable data, permit us a fair and reliable comparison between two different data analysis approach. Moreover, the data from the sensory glove and the IMUs are collected and synchronized, providing real-time data-fusion from both sensory glove and IMUs.

As a meaningful further consideration, our database was constructed with signs performed by trainees rather than expert native signers, which adds a sort of “robustness” to the tested classification algorithms. Indeed, a non-native speaker typically repeats a specific gesture with minor precision with respect to a native speaker. Thus, the analysis applies to more dispersed values, thus enabling an expected better performance in case of native-speaker better-accuracy gestures (to be demonstrated in a future work).

Further research includes improvements on the *k*-NN and DTW model using an efficient reduction of the data dimensionality. In this way, better and faster results might be obtained, which in turn may also allow us to recognize more gestures for real-time applications. Finally, both models need to be tested at classifying more classes for different sign languages and for the recognition of consecutive sentences.

## Figures and Tables

**Table 1:** Confusion matrix for the k-NN and DTW classification model.

**Google**	135; 9.6%	0; 0%	0; 0%	0; 0%	1; 0.1%	0; 0%	0; 0%	13; 0.9%	2; 0.1%	0; 0%	89.4%
**Internet**	0; 0%	132; 9.4%	0; 0%	0; 0%	0; 0%	0; 0%	0; 0%	4; 0.3%	0; 0%	0; 0%	97.1%
**Jogging**	2; 0.1%	0; 0.0%	140; 10%	0; 0%	8; 0%	0; 0%	0; 0%	0; 0%	0; 0%	0; 0%	93.3%
**Pizza**	0; 0%	0; 0%	0; 0%	140; 10%	0; 0%	0; 0%	0; 0%	0; 0%	0; 0%	0; 0%	100%
**Television**	3; 0.2%	0; 0.0%	0; 0%	0; 0.0%	131; 9.4%	0; 0%	0; 0%	0; 0%	0; 0%	1; 0.1%	97.0%
**Twitter**	0; 0%	0; 0%	0; 0%	0; 0%	0; 0%	140; 10%	0; 0%	0; 0%	0; 0%	0; 0.0%	100%
**Ciao**	0; 0%	0; 0%	0; 0%	0; 0%	0; 0%	0; 0%	139; 9.9%	0; 0%	0; 0%	0; 0%	100%
**Cose**	0; 0%	0; 0%	0; 0%	0; 0%	0; 0%	0; 0%	0; 0%	120; 8.6%	0; 0%	0; 0%	100%
**Grazie**	0; 0%	8; 0.6%	0; 0%	0; 0%	0; 0%	0; 0%	1; 0.1%	3; 0.2%	138; 9.9%	1; 0.1%	91.4%
**Maestra**	0; 0%	0; 0%	0; 0%	0; 0%	0; 0%	0; 0%	0; 0%	0; 0%	0; 0%	138; 9.9%	100%;
**SENSITIVITY**	96.4%	94.3%	100%	100%	93.6%	100%	99.3%	85.7%	98.6%	98.6%	96.6%
	**Google**	**Internet**	**Jogging**	**Pizza**	**Television**	**Twitter**	**Ciao**	**Cose**	**Grazie**	**Maestra**	**PRECISION**

**Table 2:** Confusion matrix for the CNN classification model.

**Google**	139; 9.9%	0; 0%	1; 0.1%	0; 0%	0; 0%	0; 0%	0; 0%	0; 0%	1; 0.1%	0; 0%	98.6%
**Internet**	0; 0%	139; 9.9%	0; 0%	0; 0%	0; 0%	0; 0%	1; 0.1%	0; 0%	0; 0%	1; 0.1%	98.6%
**Jogging**	0; 0%	0; 0%	135; 9.6%	1; 0.1%	0; 0%	1; 0.1%	2; 0.1%	2; 0.1%	1; 0.1%	0; 0%	95.1%
**Pizza**	0; 0%	0; 0%	0; 0%	138; 9.9%	0; 0%	0; 0%	0; 0%	0; 0%	0; 0%	0; 0%	100%
**Television**	0; 0%	0; 0%	0; 0%	0; 0%	140; 10.0%	0; 0%	0; 0%	0; 0%	0; 0%	0; 0%	100%
**Twitter**	0; 0%	0; 0%	0; 0%	1; 0.1%	0; 0%	139; 9.9%	0; 0%	0; 0%	0; 0%	0; 0%	99.3%
**Ciao**	0; 0%	0; 0%	2; 0.1%	0; 0%	0; 0%	0; 0%	133; 9.5%	0; 0%	0; 0%	0; 0%	98.5%
**Cose**	1; 0.1%	0; 0%	0; 0%	0; 0%	0; 0%	0; 0%	0; 0%	137; 9.8%	1; 0.1%	2; 0.1%	97.2%
**Grazie**	0; 0%	0; 0%	0; 0%	0; 0%	0; 0%	0; 0%	0; 0%	1; 0.1%	135; 9.6%	0; 0%	99.3%
**Maestra**	0; 0%	1; 0.1%	2; 0.1%	0; 0%	0; 0%	0; 0%	4; 0.3%	0; 0%	2; 0.1%	137; 9.8%	93.8%
**SENSITIVITY**	99.3%	99.3%	96.4%	98.6%	100%	99.3%;	95.0%	97.9%	96.4%	97.9%	98.0%
	**Google**	**Internet**	**Jogging**	**Pizza**	**Television**	**Twitter**	**Ciao**	**Cose**	**Grazie**	**Maestra**	**PRECISION**

**Table 3:** Confusion matrix for the CNN classification model.

Reference	Sensor(s)	Signers, Signs, Repetitions	Classifier	Accuracy m ± s [%]
Mohandes et al., 1996 [16]	PowerGlove	n/a, 10, 20	SVM	90 ± 10
Mohandes and Deriche, 2013 [18]	CyberGloves	1, 100, 20	LDA + MD	96.2 ± 0.78
Tubaiz et al., 2015 [19]	DG5 - VHand	1, 40, 10	MKNN	82 ± 4.88
Abualola et al., 2016 [20]	AcceleGlove + skeleton	17, 1, 30	CTM	98 ± n/a
Lu et al., 2016 [22]	YoBuGlove	n/a, 10, n/a	ELM-kernel SVM	89.59 ± n/a 83.65 ± n/a
Saengsri et al., 2012 [23]	5DTGlove + tracker	1, 16, 4	ENN	94.44 ± n/a
Silva et al., 2017 [24]	Glove + IMU	1, 26, 100	ANN	95.8 ± n/a
Our work	HitegGlove + Movit G1 IMU	7, 10, 100	kNN + DTW CNN	96.6 ± 3.4 98 ± 2.0
